# Prevalence and severity of anxiety in cancer patients: results from a multi-center cohort study in Germany

**DOI:** 10.1007/s00432-023-04600-w

**Published:** 2023-02-09

**Authors:** Ute Goerling, Andreas Hinz, Uwe Koch-Gromus, Julia Marie Hufeld, Peter Esser, Anja Mehnert-Theuerkauf

**Affiliations:** 1 Charité–Universitätsmedizin Berlin, corporate member of Freie Universität Berlin, Humboldt-Universität zu Berlin, and Berlin Institute of Health, Charité Comprehensive Cancer Center, Charitéplatz 1, 10117 Berlin, Germany; 2 Department of Medical Psychology and Medical Sociology, University Medical Center Leipzig, Leipzig, Germany; 3 Department of Medical Psychology, University Medical Center Hamburg-Eppendorf, Hamburg, Germany

**Keywords:** Anxiety, Cancer, Oncology, Anxiety symptoms, GAD-7

## Abstract

**Purpose:**

Anxiety is an accompanying symptom in cancer patients that can have a negative impact on patients. The aim of the present analyses is to determine the prevalence of anxiety, taking into account sociodemographic and medical variables, and to determine the odds ratio for the occurrence of anxiety in cancer patients compared to general population.

**Methods:**

In this secondary analyses, we included 4,020 adult cancer patients during and after treatment from a multi-center epidemiological study from 5 regions in Germany in different treatment settings and a comparison group consisting of 10,000 people from the general population in Germany. Anxiety was measured with the Generalized Anxiety Disorder (GAD-7) questionnaire. In multivariate analyses adjusted for age and sex, we calculated the odds of being anxious.

**Results:**

The prevalence of anxiety was observed to be 13.8% (GAD-7 ≥ 10). The level of anxiety was significant higher for patients in rehabilitation, compared to patients during inpatient and outpatient treatment (*p* = .013). Comparison with the general population yielded a 2.7-fold increased risk for anxiety among cancer patients (95% CI 2.4–3.1; *p* < .001). Patients with bladder cancer (OR, 5.3; 95% CI 3.0–9.4) and testicular cancer (OR, 5.0; 95% CI 2.1–12.1) showed the highest risk of having high levels of anxiety.

**Conclusion:**

The results highlight the importance of identifying anxiety in cancer patients.

## Introduction

A critical life event may increase the risk for psychological distress and anxiety (Hassanzadeh et al. 2017). Therefore, anxiety is also a well-known accompanying phenomenon in cancer patients (Kapfhammer 2015; Bates et al. 2017; Pitman et al. 2018). A meta-analysis of studies from different countries found a prevalence of anxiety disorders in 10.3% (95 CI 5.1–17.0) of patients for hematology and oncology (Mitchell et al. 2011). This same meta-analysis demonstrated anxiety disorders in 9.8% (95% CI 6.8–13.2) of palliative patients. Another review reported both a pooled adjusted 4-week prevalence of anxiety disorders in cancer patients of 13.5% (95% CI 7.1–24.3) based on 10 studies and a lifetime prevalence of 30.5% (95% CI 28.0–33.0) based on 2 studies for Germany (Vehling et al. 2012). Both reviews integrated studies that assessed anxiety disorders by structured clinical interviews according to Diagnostic and Statistical Manual of Mental Disorders (DSM) or International Statistical Classification of Diseases and Health-Related Problems (ICD-10). It is known from other studies that results depend on the method chosen and vary accordingly (Krebber et al. 2014). In contrast, studies using validated questionnaires for self-assessment of anxiety showed different results. A study conducted in Utrecht observed anxiety symptoms in 22% of 2,144 inpatients (Van Den Brekel et al. 2020). In patients treated exclusively with symptomatic palliative care, the prevalence raised up to 36%. Predictors proved to be female gender, younger age, depressed mood, sleeping problems, and dyspnea. Maass et al. (2015) concluded from a review of studies in women with breast cancer that the prevalence of anxiety ranges from 17.9 to 33.3% and remains so in breast cancer survivors. Anxiety in patients with cancer in low- and lower-middle-income countries was addressed by authors in another review (Walker et al. 2021). They observed a prevalence between 5.6 and 89.2%. These were often associated with advanced disease and low levels of education. However, these results have to be considered cautiously against the background of different questionnaires and different thresholds.

Why is anxiety a non-negligible factor in the context of oncological diseases? Anxiety and a correspondingly elevated physiological level of arousal increase functions that can be influenced by the autonomic nervous system, such as pain, nausea and vomiting. This in turn can lead to a renewed increase in anxiety. Anxiety can lead to behavioral paralysis and patients are unable to apply adequate coping strategies. Therefore, anxiety can affect compliance with treatment (Housman et al. 2021). A correlation between anxiety and increased side effects during chemotherapy could be observed (Mahdizadeh et al. 2019). A study in cancer patients on oral targeted or chemotherapy reported an association between fatigue and anxiety (Poort et al. 2020). Furthermore, it could be shown that anxiety influence the functional outcome (Dinesh et al. 2021). A review of international studies revealed that clinically diagnosed anxiety disorders and anxiety defined by scales were related with higher cancer-specific mortality as well as poorer survival (Wang et al. 2020). A study in Germany examined 436 female patients with breast cancer concluded that increased anxiety was associated with greater dissatisfaction with information both at baseline and 12 months follow up (Faller et al. 2017). Another study from Germany showed a similar result. Here, in a multi-center longitudinal study, 1,398 patients with various cancer diagnoses were surveyed regarding their experience of anxiety at three different points in time (baseline, 6 months and 12 months follow up). As a result, it was shown that increased anxiety at all time points was associated with greater dissatisfaction with information perceived (Goerling et al. 2020). Analyses of the prevalence of anxiety in long-term cancer survivors yielded 17.9% (95% CI 12.8–23.6) (Mitchell et al. 2013). Out of 1154 survivors, 21% reported a score of ≥ 8 on the anxiety scale measured with the Hospital Anxiety and Depression Scale (HADS) 12 months after diagnosis (Boyes et al. 2013). Anxiety has a negative impact on quality of life after cancer (Saevarsdottir et al. 2010).

Many previous studies have targeted patient populations with common tumor diseases such as breast or prostate cancer or specific stages of cancer treatment. We therefore aim to estimate the prevalence of anxiety based on a large multi-center epidemiological study with multiple tumor locations. We analyze differences between individual tumor entities during and after treatment and we compare the anxiety of these patients with the age- and gender-adjusted general population.

## Methods

Here, we present data from a secondary analysis of a multi-center epidemiological cross-sectional study where we investigated the prevalence of mental disorders, psychosocial distress and need for psychosocial support in cancer patients (Mehnert et al. 2014). The sample is composed of oncology patients receiving treatment in both inpatient and outpatient settings. We also included patients in cancer rehabilitation. Patients with malignant tumor across all entities and disease stages were included, and stratified by nationwide incidence of cancer diagnoses. The recruitment took place consecutively from acute care hospitals, outpatient cancer care facilities, and cancer rehabilitation centers in Germany. All patients provided written informed consent. The Ethics Committees of all participating centers obtained positive ethical approval. The study complied with the Declaration of Helsinki as well as the terms of data protection and privacy laws. The study protocol was described in detail elsewhere (Mehnert et al. 2012).

### General population data

To compare anxiety from cancer patients with the general population, we used data from a large population-based study, including 10,000 people in Germany (Loeffler et al. 2015; Hinz et al. 2017). This study contained an age- and gender-stratified random selection of inhabitants.

### Measures

For the demographic variables age, sex, partnership, years of education, and work situation of cancer patients were documented. Medical data were obtained from medical records and included cancer diagnosis (ICD-10, WHO; 2004), tumor stage (UICC TNM classification; Sobin et al. 2009), metastases, cancer disease condition, treatment intention (curative/palliative), and completed treatment (surgery, radiation, chemotherapy, hormone treatment). Furthermore, we obtained the performance status (ECOG; Oken et al. 1982). The score can range from 0 (asymptomatic) to 4 (bedbound).

Anxiety was measured using the German version of GAD-7 (Spitzer et al. 2006; Löwe et al. 2008). This is a one-dimensional instrument created to detect symptoms of generalized anxiety disorders defined in the DSM-IV (APA 2000), and has excellent reliability, as well as criterion, construct, factorial, and procedural validity. Core symptoms within the past two weeks were queried with seven items, which are scored on a four-point Likert scale rated from 0 (not at all) to 3 (nearly every day). The total GAD-7 score can range from 0 to 21. A score of 0 to 4 indicates the absence of generalized anxiety disorder, score of 5 to 9 represents mild, score of 10 to 14 represents moderate, and score of 15 and higher represents serve anxiety symptom levels (Löwe et al. 2008).

### Statistical analyses

We compared participants and non-participants in multiple logistic regression models according adjusted differences in age, sex, education, treatment setting, cancer type, and study center.

We calculated frequencies as well as means and standard deviations, as appropriate. Sociodemographic and medical groups were compared in terms of anxiety symptoms according to the GAD-7 mean score with one-way analysis of variance (ANOVA).

To dichotomize the scores and divide them into “anxious” and “non-anxious”, the cut of ≥ 10 was chosen, following the recommendation of Andersen et al. (2014). To compare cancer patients with different types of cancer during and after treatment with the general population, we performed multivariate analyses using logistic regression. We adjusted odds ratios for confounders by including ages and sex in the regression model. Gender-specific tumors were the exceptions. Women with breast cancer and female genital tumors were compared only with women from general population, and patients with testicular cancer and prostate cancer were compared only with men. An adjustment was made here according to age. A *p* value < 0.05 was considered statistically significant. Analyses were performed using SPSS version 27 (SPSS Inc., Chicago, III, USA).

## Results

### Characteristics of the sample of cancer patients

Out of 5,889 eligible cancer patients, 4,020 (69%) participated. Participants in this group were younger (*p* < 0.001), more educated (*p* < 0.001) and were more likely to be recruited from a cancer rehabilitation center (*p* < 0.001) than during treatment from acute care hospitals. We found no sex difference in study participation (*p* < 0.10). Participants’ mean age was 58 years (SD = 11). Mean time since diagnosis was 13.5 months (SD = 25). Demographic and medical sample characteristics are presented in Table 1.

**Table 1:** Sociodemographic and medical sample characteristics of anxiety (GAD-7) in cancer patients in Germany

	Total sample		Anxiety (GAD-7 ≥ 10)		GAD-7		
	*N*	%	*N*	%	*M*	SD	*p*
Sex							
Woman	2068	51.4	329	16.8	5.70	4.16	< 0.001
Men	1952	48.6	194	10.7	4.57	3.87	
Age category^a^							
18–35	151	3.8	26	18.4	5.6	4.02	< 0.001
36–45	385	9.7	74	20.1	6.0	4.34	
46–55	897	22.5	157	18.2	5.9	4.23	
56–65	1265	31.8	151	12.8	5.0	4.05	
66–75	1286	32.3	110	9.2	4.4	3.71	
In a relationship^a^							
Yes	2904	80.7	377	13.2	5.1	4.22	0.021
No	696	19.3	113	16.7	5.5	4.02	
Education							
≤ 10 years	2456	61.1	343	14.3	5.2	4.16	0.459
> 10 years	1564	38.9	180	13.8	5.1	3.89	
Work situation^a^							
Employed	1569	42.5	223	14.1	5.3	3.99	< 0.001
Unemployed	208	5.6	44	21.3	6.1	3.99	
Retired	1732	46.9	214	12.7	4.9	4.11	
Housewife/househusband	187	5.1	20	11.0	4.7	3.75	
Cancer care setting							
Inpatients oncology ward	1735	43.2	213	13.3	5.1	4.04	0.013
Outpatient oncology clinics	1324	32.9	162	13.0	5.0	3.96	
Cancer rehabilitation	961	23.9	148	15.9	5.5	4.23	
Cancer type							
Testis	36	0.9	7	20.6	5.4	4.53	< 0.001
Bladder	90	2.2	16	19.8	5.4	4.16	
Female genital organs	317	7.9	53	17.5	5.9	4.17	
Soft tissue	39	1.0	6	17.1	6.2	4.51	
Lung	331	8.2	49	16.8	5.2	4.41	
Thyroid	25	0.6	4	16.7	5.6	3.88	
Breast	906	22.5	140	16.2	5.5	4.08	
Head and neck	127	3.2	18	15.4	4.9	4.20	
Stomach/esophagus	146	3.6	21	14.9	5.6	4.40	
Brain	62	1.5	8	13.8	5.3	4.01	
Hematological	305	7.6	40	13.7	5.4	4.04	
Malignant melanoma	67	1.7	9	13.4	5.5	4.36	
Colon/rectum	510	12.7	59	12.4	4.9	3.88	
Kidney/urinary tract	131	3.3	15	12.3	4.7	4.35	
Hepatobiliary	52	1.3	6	11.8	5.0	4.16	
Prostate	637	15.8	48	8.1	4.1	3.54	
Pancreas	82	2.0	6	7.7	4.8	3.46	
Other	157	3.9	18	12.2	5.2	4.13	
Cancer disease condition^a^							
In remission	1539	39.4	200	13.6	5.1	3.98	0.683
Not in remission	2365	60.6	310	14.1	5.2	4.12	
Tumor stage /UICC TNM)^a^							
I	539	13.6	71	13.7	5.2	3.89	0.459
II	700	17.7	93	14.2	5.1	4.19	
III	546	13.8	63	12.2	4.8	3.80	
IV	889	22.4	124	15.2	5.2	4.19	
Metastases^a^							
Yes	870	21.9	119	14.9	5.3	4.19	0.491
No	2400	60.4	298	13.2	5.1	3.98	
Treatment intention^a^							
Curative	2396	59.6	292	12.8	5.0	3.95	0.008
Palliative	926	23.0	140	16.4	5.6	4.35	
Surgery^a^							
Yes	1039	26.8	388	14.4	5.2	4.10	0.065
No	2838	73.2	121	12.6	4.9	3.98	
Radiation^a^							
Yes	1762	45.1	239	14.2	5.1	4.09	0.949
No	2145	54.9	272	13.6	5.1	4.05	
Chemotherapy^a^							
Yes	2023	51.6	290	15.3	5.3	4.17	0.001
No	1896	48.4	221	12.4	4.9	3.93	
Hormone therapy^a^							
Yes	481	87.7	67	14.5	5.2	3.95	0.691
No	3420	12.3	444	13.8	5.1	4.09	
ECOG performance status^a^							
0	1900	49.2	200	11.1	4.7	3.83	< 0.001
1	1368	35.4	218	16.8	5.5	4.24	
2	465	12.0	63	14.8	5.5	4.02	
3	113	2.9	20	21.3	6.4	4.59	
4	13	0.3	3	25.0	6.3	3.44	

*GAD-7*, Generalized Anxiety Disorder–German version, *N* number of patients, *M* mean, *SD* standard deviation, ^***a***^Reduced sample size due to missing data, *p* value based on one-way ANOVA

### Anxiety in cancer patients

**Fig. 1:**
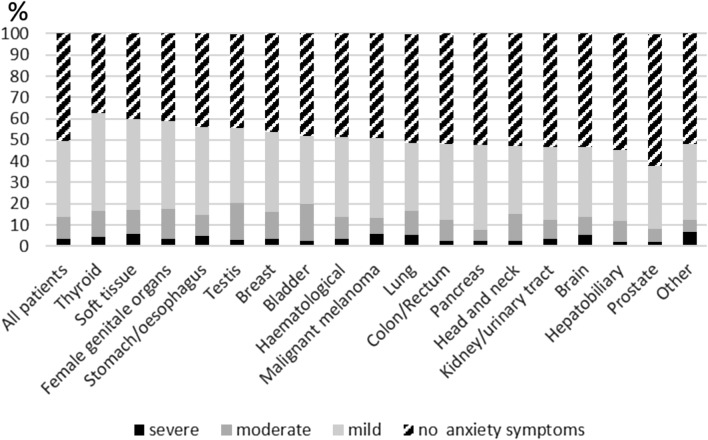
Severity of anxiety (GAD-7 scores) by tumor entities in Germany. *GAD-7* Generalized Anxiety Disorder–German version

Out of all cancer patients, 13.8% showed elevated anxiety (GAD ≥ 10), while severe anxiety (GAD ≥ 15) were relatively rare across tumor entities (Fig. 1). A significantly greater proportion of women compared with men reported anxiety (16.8 vs. 10.7%, *p* < 0.001). The highest prevalence of anxiety was observed in patients in the age category 36–45 years (20.1%), in single patients (13.2%), or in unemployed patients (21.3%). Even if not significant, a trend toward a greater proportion of patients in rehabilitation reported anxiety compared with patients during treatment (15.9 vs. 13.3% resp. 13.0%, *p* = 0.102). Nevertheless, the mean expression of anxiety is significantly greater in cancer rehabilitation than during treatment (5.5 vs. 5.1 resp. 5.0, *p* = 0.013). Furthermore, regarding the type of therapy, patients reported more anxiety after chemotherapy. The prevalence of anxiety was most evident in patients with testicular cancer, followed by bladder cancer, female genital tumors, patients with soft tissue tumors, lung cancer, and thyroid cancer. Patients with prostate carcinoma or pancreatic carcinoma reported relatively little anxiety. All results are listed in Table 1.

### Anxiety in cancer patients compared to general population

The two samples do not differ significantly in terms of age and sex. The proportion of anxiety in cancer patients is significantly higher at 13.8% compared to 5.7% in the general population (*p* < 0.001). Multivariate logistic regression analyses adjusted for age and sex showed significantly higher odds for anxiety in cancer patients (OR, 2.7; 95% CI 2.4–3.1; *p* < 0.001). We found similar results for the different treatment settings. Thus, the risk of anxiety was significantly higher in patients during cancer rehabilitation than in the general population (OR, 3.1; 95% CI 2.6 – 3.8; *p* < 0.001). It was increased 2.8-fold (95% CI 2.3–3.3; *p* < 0.001) during inpatient treatment and 2.4-fold (95% CI 2.0–2.9; *p* < 0.001) during outpatient treatment compared to the general population. An increased risk for anxiety was revealed in all other tumor entities except for patients with pancreatic carcinoma. Particularly, patients with bladder cancer were most likely to report anxiety with odds more than 5 times higher than those in general population (OR, 5.3; 95% CI 3.0–9.4). We observed a significantly increased risk for anxiety in testicular cancer patients (OR, 5.0; 95% CI 2.1–12.1). All results are listed in Table 2.

**Table 2:** Odds ratios for risk for anxiety in cancer patients (*N* = 4.020) compared to general population sample (*N* = 10.000) in Germany

	Anxiety (GAD-7 ≥ 10)			
	OR	95% CI		*p*
		LB	UB	
All cancer patients	2.7^a^	2.4	3.1	< 0.001
Setting				
Cancer rehabilitation	3.1^a^	2.6	3.8	< 0.001
Inpatient oncology wards	2.8^a^	2.3	3.3	< 0.001
Outpatient oncology clinic	2.4^a^	2.0	2.9	< 0.001
Cancer type				
Bladder	5.3^a^	3.0	9.4	< 0.001
Testis	5.0^b^	2.1	12.1	< 0.001
Lung	3.9^a^	2.8	5.4	< 0.001
Soft tissue	3.6^a^	1.5	8.9	0.004
Head and neck	3.5^a^	2.1	5.9	< 0.001
Stomach/ esophagus	3.5^a^	2.2	5.6	< 0.001
Thyroid	3.1^a^	1.0	9.1	0.043
Malignant melanoma	2.8^a^	1.3	5.6	0.005
Female genital organ	2.7^c^	2.0	3.7	< 0.001
Brain	2.6^a^	1.2	5.5	0.015
Kidney/urinary tract	2.6^a^	1.5	4.6	0.001
Hematological	2.6^a^	1.8	3.7	< 0.001
Colon/ rectum	2.6^a^	1.9	3.4	< 0.001
Breast	2.5^c^	2.0	3.1	< 0.001
Hepatobiliary	2.5^a^	1.0	5.9	0.038
Prostate	2.4^b^	1.7	3.3	< 0.001
Other	2.4^a^	1.5	4.0	0.001
Pancreas	1.5^a^	0.6	3.5	0.342

Confounders adjusted for ^a^age and sex, ^b^men, adjusted for age, ^c^women adjusted for age

OR, odds ratio; GAD-7, Generalized Anxiety Disorder–German version, 95% *CI* 95% confidence interval, *LB* lower boundary, *UB* upper Boundary

## Discussion

In this study, we aimed to measure and analyze the prevalence of anxiety in cancer patients taking into account sociodemographic and medical variables. We further compared levels of anxiety among cancer patients to the general population.

### Anxiety in cancer patients

In our study, anxiety was found in almost 14% of cancer patients. This result is similar to the results of a cross-sectional study in which data on anxiety were collected with the Brief Symptom Inventory. The prevalence of anxiety in outpatients was about 12% (Brintzenhofe-Szoc et al. 2009). Another study, also surveyed self-reported anxiety, reported more frequently anxiety (22%) (Van Den Brekel et al. 2020). Because this study surveyed only oncology patients at the time of hospital admission, uncertainty about the subsequent course and upcoming treatments could be an indication of higher level of anxiety. However, this difference remains even when anxiety levels are considered with our inpatients (13%).

Another study with a large number of participants reported 19% of cancer patients showed clinical level of self-reported anxiety (Linden et al. 2012). Nevertheless, the measurement instruments used were different. The survey time points are also not comparable. For example, Linden et al. (2012) collected data before the first visit to a cancer center and thus before the start of treatment. In Brazil, outpatient oncology patients were found to have anxiety symptoms in 21.4%, also measured by the GAD-7 (Polidoro Lima and Osório 2014). Nevertheless, comparability with our study results is limited because the authors did not reported cut-off values used. Our results included samples from different treatment settings. However, the frequencies for anxiety did not differ between during treatment (inpatient and outpatient), and in rehabilitation. In contrast, however, a higher level of anxiety was observed after the end of therapy. Presumably, anxiety level is high before the start of treatment, because patients do not yet know what they will have to face. Anxiety after completing treatment could also include fear of disease recurrence. Yi and Syrjala (2017) stated, that patients after treatment report feeling alone or even abandoned following the intensive support provided during their treatment, and survivors often anxious and hypervigilant to physical sensations. A study from Germany observed that 9% of survivors reported moderate to severe anxiety 5 years after their cancer (Götze et al. 2020).

In our study, women reported anxiety more frequently and at higher levels than men. These findings generally fit into the literature that women reported more anxiety (Linden et al. 2012, Van Den Brekel et al. 2020) and distress related to cancer (Carlson et al. 2019; Herschbach et al. 2020). It seems that men focus more on physical problems while women tend to report emotional concerns (Loscalzo et al. 2018). We observed the highest proportion of anxiety in patients in the age group between 36 and 45 years. We suspect that this could be related to the current phase of life. Possibly the focus here is on anxiety about small children and the family. With increasing age, the proportion of patients who reported anxiety decreased. This finding is consistent with the results of the study by Meier et al. (2020) in which patients with hematologic cancers aged 70 and older reported low mean anxiety.

Both Linden et al. (2012) and Polidoro Lima and Osório (2014) demonstrated that anxiety was highest in women with gynecologic tumors. In our sample, patients with testicular tumor, bladder cancer, and soft tissue tumors had the greatest anxiety. However, these entities were not included in the sample in the two studies cited. Following this, women with gynecologic tumors also had very high anxiety in our sample. In contrast, others found increased anxiety in patients with lymphoma as well as breast cancer (Brintzenhofe-Szoc et al. 2009). These patient groups also reported a high level of anxiety in our study.

### Anxiety in cancer patients compared to general population

Comparison of the prevalence of anxiety in cancer patients with the general population in Germany yielded a 2.7-fold increased risk for anxiety in cancer patients. With a 3.1-fold increased risk, patients in rehabilitation are even higher. We suspect that at this point the fear of late effects of the cancer and recurrence of the disease may increase. Perhaps patients who have completed treatment will become aware of their situation during rehabilitation. Even 5 years after the disease, a significantly higher level of anxiety, measured with GAD-7, was observed in cancer survivors in the age group up to 60 years in Germany compared to the general population (Götze et al. 2020).

Most of the tumor entities we studied are also above the general risk. Patients with testicular or lung cancer and, above all, bladder cancer were clearly at higher risk. These results are partly in the line with other studies. A systematic review indicates that patients with lung cancer experienced significantly more symptom distress than other cancer patients (Cooley 2000). A study from Scotland of 170 patients with lung cancer identified also an increased self-rated anxiety in over half of the patients (Buchanan et al. 2010). However, only patients in a palliative situation were included there. Regarding the association of bladder cancer and anxiety, another review showed very different results. The prevalence was 9.8% before treatment and ranged from 12.5 to 23% after treatment in Europe (Vartolomei et al. 2018).

Compared with the general population, patients with prostate cancer reported the least anxiety. One explanation could lie in the gender specificity of this condition, as we were able to show that men generally reported less anxiety than women did (Loscalzo and Clark 2018). Sánchez Sánchez et al. (2020) reported a prevalence of anxiety of 14.1% in 184 patients with prostate cancer as measured by the Hospital Anxiety and Depression Scale in Spain. In this regard, higher level of anxiety also appeared to be associated with multimodal treatment.

Surprisingly, we did not find a significant difference in patients with pancreatic cancer. A relationship with depressiveness does not seem to exist here, then this group of patients had a 7.8-fold increased risk of developing depression (Hartung et al. 2017). With a prevalence of anxiety of 7.7%, these patients are clearly below the prevalence of cancer patients in general in our analysis. A statistically significant difference to the general population could not be observed. Again, this could also be due to the small sample size. At this point, our results are contradictory to results of other studies that showed more anxiety in patients with pancreatic cancer (Kenner 2018). Certainly, social desirability of the responses, as well as different tumor stages and inconsistent measurement instruments, could be decisive here as well and complicate the comparison.

### Strengths and limitations

Strengths of the study are the multi-center design, especially the inclusion of patients with different tumor entities with different settings. The participation rate of 69% can be considered very high. Another advantage of our analyses is the comparison with prevalence data for anxiety from a large German general population. An important criterion for this is the use of the same measurement instrument and the same cut-off value. Nevertheless, there are some sources of bias to discuss that could affect the generalizability of the results. The most common reason given for non-participation was "too burdened". Perhaps these patients in particular were very anxious, also of psychological stigmatization. One more limitation mainly to be seen is the cross-sectional study. Future studies should prefer a measurement over time as a longitudinal section. In our sample, we did not check how many or which patients had a second tumor. With respect to the sample of the general population, we also cannot make any statements regarding possible comorbidities and the level of education. Statements regarding comparisons in terms of tumor entities must also be viewed with caution, as the studies included very different samples.

## Conclusion

In our study, a high prevalence of anxiety was observed in cancer patients and a significantly higher risk compared to the general population. Since physicians and nurses very often overlook the presence of clinically relevant anxiety in oncology patients and assume an appropriate emotional response, it is important to identify anxiety in cancer patients at an early stage. These are related to disease and treatment. Also after completion the treatment, an increased level of anxiety is observed. Anxiety can negatively affect treatment adherence and have a significant impact on quality of life.

## Data Availability

The datasets generated during and/or analyzed during the current study are available from the corresponding author on reasonable request.

## References

[CR1] Andersen BL, DeRubeis RJ, Berman BS, Gruman J, Champion VL, Massie MJ, Holland JC, Partridge AH, Bak K, Somerfield MR, Rowland JH (2014). Screening, assessment, and care of anxiety and depressive symptoms in adults with cancer: an american society of clinical oncology guideline adaptation. J Clin Oncol.

[CR2] APA (2000). Diagnostic and Statistical Manual of Mental Disorders DSM-IV-TR. Washington, DC., American Psychiatric Association.

[CR3] Bates GE, Mostel JL, Hesdorffer M (2017). Cancer-Related Anxiety. JAMA. Oncol.

[CR4] Boyes AW, Girgis A, D'Este CA, Zucca AC, Lecathelinais C, Carey ML (2013). Prevalence and predictors of the short-term trajectory of anxiety and depression in the first year after a cancer diagnosis: a population-based longitudinal study. J Clin Oncol.

[CR6] Brintzenhofe-Szoc KM, Levin TT, Li Y, Kissane DW, Zabora JR (2009). Mixed anxiety/depression symptoms in a large cancer cohort: prevalence by cancer type. Psychosomatics.

[CR7] Buchanan D, Milroy R, Baker L, Thompson AM, Levack PA (2010). Perceptions of anxiety in lung cancer patients and their support network. Support Care Cancer.

[CR8] Carlson LE, Zelinski EL, Toivonen KI, Sundstrom L, Jobin CT, Damaskos P, Zebrack B (2019). Prevalence of psychosocial distress in cancer patients across 55 north american cancer centers. J Psychosoc Oncol.

[CR9] Cooley ME (2000). Symptoms in adults with lung cancer a systematic research review. J Pain Symptom Man.

[CR10] Dinesh AA, Helena Pagani SPS, Brunckhorst O, Dasgupta P, Ahmed K (2021). Anxiety, depression and urological cancer outcomes: a systematic review. Urol Oncol.

[CR11] Faller H, Strahl A, Richard M, Niehues C, Meng K (2017). The prospective relationship between satisfaction with information and symptoms of depression and anxiety in breast cancer: A structural equation modeling analysis. Psycho-Oncol.

[CR12] Goerling U, Faller H, Hornemann B, Hönig K, Bergelt C, Maatouk I, Stein B, Teufel M, Erim Y, Geiser F, Niecke A, Senf B, Wickert M, Büttner-Teleaga A, Weis J (2020). Information needs in cancer patients across the disease trajectory A prospective study. Patient Edu Counsel..

[CR13] Götze H, Friedrich M, Taubenheim S, Dietz A, Lordick F, Mehnert A (2020). Depression and anxiety in long-term survivors 5 and 10 years after cancer diagnosis. Support Care Cancer.

[CR14] Hartung TJ, Brähler E, Faller H, Härter M, Hinz A, Johansen C, Keller M, Koch U, Schulz H, Weis J, Mehnert A (2017). The risk of being depressed is significantly higher in cancer patients than in the general population: Prevalence and severity of depressive symptoms across major cancer types. Eur J Cancer.

[CR15] Hassanzadeh A, Heidari Z, Feizi A, Hassanzadeh Keshteli A, Roohafza H, Afshar H, Adibi P (2017). Association of Stressful Life Events with Psychological Problems: A Large-Scale Community-Based Study Using Grouped Outcomes Latent Factor Regression with Latent Predictors. Comput Math Method Med..

[CR16] Herschbach P, Britzelmeir I, Dinkel A, Giesler JM, Herkommer K, Nest A, Pichler T, Reichelt R, Tanzer-Küntzer S, Weis J, Marten-Mittag B (2020). Distress in cancer patients: Who are the main groups at risk?. Psycho-Oncol.

[CR17] Hinz A, Klein AM, Brähler E, Glaesmer H, Luck T, Riedel-Heller SG, Wirkner K, Hilbert A (2017). Psychometric evaluation of the Generalized Anxiety Disorder Screener GAD-7, based on a large German general population sample. J Affective Disorder..

[CR18] Housman B, Flores R, Lee DS (2021). Narrative review of anxiety and depression in patients with esophageal cancer: underappreciated and undertreated. J Thorac Dis.

[CR19] Kapfhammer HP (2015). Comorbid depressive and anxiety disorders in patients with cancer. Nervenarzt.

[CR20] Kenner BJ (2018). Early detection of pancreatic cancer: the role of depression and anxiety as a precursor for disease. Pancreas.

[CR21] Krebber AM, Buffart LM, Kleijn G, Riepma IC, de Bree R, Leemans CR, Becker A, Brug J, van Straten A, Cuijpers P, Verdonck-de Leeuw IM (2014). Prevalence of depression in cancer patients: a meta-analysis of diagnostic interviews and self-report instruments. Psychooncology.

[CR22] Linden W, Vodermaier A, Mackenzie R, Greig D (2012). Anxiety and depression after cancer diagnosis: prevalence rates by cancer type, gender, and age. J Affect Disord.

[CR23] Loeffler M, Engel C, Ahnert P, Alfermann D, Arelin K, Baber R, Beutner F, Binder H, Brähler E, Burkhardt R, Ceglarek U, Enzenbach C, Fuchs M, Glaesmer H, Girlich F, Hagendorff A, Häntzsch M, Hegerl U, Henger S, Hensch T, Hinz A, Holzendorf V, Husser D, Kersting A, Kiel A, Kirsten T, Kratzsch J, Krohn K, Luck T, Melzer S, Netto J, Nüchter M, Raschpichler M, Rauscher FG, Riedel-Heller SG, Sander C, Scholz M, Schönknecht P, Schroeter ML, Simon JC, Speer R, Stäker J, Stein R, Stöbel-Richter Y, Stumvoll M, Tarnok A, Teren A, Teupser D, Then FS, Tönjes A, Treudler R, Villringer A, Weissgerber A, Wiedemann P, Zachariae S, Wirkner K, Thiery J (2015). The LIFE-Adult-Study: objectives and design of a population-based cohort study with 10,000 deeply phenotyped adults in Germany. BMC Public Health.

[CR24] Loscalzo M, Clark K (2018). Gender opportunities in psychosocial oncology. Recent Results Cancer Res.

[CR25] Löwe B, Decker O, Müller S, Brähler E, Schellberg D, Herzog W, Herzberg PY (2008). Validation and standardization of the generalized anxiety disorder screener (GAD-7) in the general population. Med Care.

[CR26] Maass SW, Roorda C, Berendsen AJ, Verhaak PF, de Bock GH (2015). The prevalence of long-term symptoms of depression and anxiety after breast cancer treatment: a systematic review. Maturitas.

[CR27] Mahdizadeh MJ, Tirgari B, Abadi O, Bahaadinbeigy K (2019). Guided imagery: reducing anxiety, depression, and selected side effects associated with chemotherapy. Clin J Oncol Nurs.

[CR28] Mehnert A, Koch U, Schulz H, Wegscheider K, Weis J, Faller H, Keller M, Brähler E, Härter M (2012). Prevalence of mental disorders, psychosocial distress and need for psychosocial support in cancer patients - study protocol of an epidemiological multi-center study. BMC Psychiat.

[CR29] Mehnert A, Brähler E, Faller H, Härter M, Keller M, Schulz H, Wegscheider K, Weis J, Boehncke A, Hund B, Reuter K, Richard M, Sehner S, Sommerfeldt S, Szalai C, Wittchen HU, Koch U (2014). Four-week prevalence of mental disorders in patients with cancer across major tumor entities. J Clin Oncol.

[CR30] Meier C, Taubenheim S, Lordick F, Mehnert-Theuerkauf A, Götze H (2020). Depression and anxiety in older patients with hematological cancer (70+) – Geriatric, social, cancer- and treatment-related associations. J Geriatric Oncol.

[CR31] Mitchell AJ, Chan M, Bhatti H, Halton M, Grassi L, Johansen C, Meader N (2011). Prevalence of depression, anxiety, and adjustment disorder in oncological, haematological, and palliative-care settings: a meta-analysis of 94 interview-based studies. Lancet Oncol.

[CR32] Mitchell AJ, Ferguson DW, Gill J, Paul J, Symonds P (2013). Depression and anxiety in long-term cancer survivors compared with spouses and healthy controls: a systematic review and meta-analysis. Lancet Oncol.

[CR33] Oken MM, Creech RH, Tormey DC, Horton J, Davis TE, McFadden ET, Carbone PP (1982). Toxicity and response criteria of the eastern cooperative oncology group. Am J Clin Oncol.

[CR34] Pitman A, Suleman S, Hyde N, Hodgkiss A (2018). Depression and anxiety in patients with cancer. BMJ.

[CR35] Polidoro Lima M, Osório FL (2014). Indicators of psychiatric disorders in different oncology specialties: a prevalence study. J Oncol.

[CR36] Poort H, Jacobs JM, Pirl WF, Temel JS, Greer JA (2020). Fatigue in patients on oral targeted or chemotherapy for cancer and associations with anxiety, depression, and quality of life. Palliat Support Care.

[CR37] Saevarsdottir T, Fridriksdottir N, Gunnarsdottir S (2010). Quality of life and symptoms of anxiety and depression of patients receiving cancer chemotherapy: longitudinal study. Cancer Nurs.

[CR38] Sánchez Sánchez E, González Baena AC, González Cáliz C, Caballero Paredes F, Moyano Calvo JL, Castiñeiras Fernández J (2020). Prevalence of anxiety and depression in prostate cancer patients and their spouses: an unaddressed reality. Prostate Cancer.

[CR39] Sobin L, Gospodarowicz M, Wittekind C (2009). TNM Classification of malignant tumours. Chichester.

[CR40] Spitzer RL, Kroenke K, Williams JB, Löwe B (2006). A brief measure for assessing generalized anxiety disorder: the GAD-7. Arch Intern Med.

[CR5] van den Brekel L, van der Baan FH, Zweers D, Koldenhof JJ, Vos JBH, de Graeff A, Witteveen PO, Teunissen S (2020). Predicting anxiety in hospitalized cancer patients. J Pain Symptom Manage.

[CR41] Vartolomei L, Ferro M, Mirone V, Shariat SF, Vartolomei MD (2018). Systematic review: depression and anxiety prevalence in bladder cancer patients. Bladder Cancer.

[CR42] Vehling S, Koch U, Ladehoff N, Schön G, Wegscheider K, Heckl U, Weis J, Mehnert A (2012). Prevalence of affective and anxiety disorders in cancer: systematic literature review and meta-analysis. Psychother Psychosom Med Psychol.

[CR43] Walker ZJ, Xue S, Jones MP, Ravindran AV (2021). Depression, anxiety, and other mental disorders in patients with cancer in low- and lower-middle-income countries: a systematic review and meta-analysis. JCO Glob Oncol.

[CR44] Wang YH, Li JQ, Shi JF, Que JY, Liu JJ, Lappin JM, Leung J, Ravindran AV, Chen WQ, Qiao YL, Shi J, Lu L, Bao YP (2020). Depression and anxiety in relation to cancer incidence and mortality: a systematic review and meta-analysis of cohort studies. Mol Psychiatry.

[CR45] WHO (2004). ICD-10: international statistical classification of diseases and related health problems : tenth revision.

[CR46] Yi JC, Syrjala KL (2017). Anxiety and depression in cancer survivors. Med Clin North Am.

